# Prostate cancer cell-derived exosomes ZNF667-AS1 reduces TGFBR1 mRNA stability to inhibit Treg expansion and DTX resistance by binding to U2AF1

**DOI:** 10.1186/s10020-024-00947-z

**Published:** 2024-10-18

**Authors:** Zhenfeng Shi, Wenjing Pu, Min Li, Mierzhayiti Aihemaitijiang, Shuo Li, Xiaoan Zhang, Bide Liu, Min Sun, Jiuzhi Li, Zhiwei Li

**Affiliations:** 1 Department of Urology Surgery Center, People’s Hospital of Xinjiang Uygur Autonomous Region, Urumqi, 830002 Xinjiang Uygur Autonomous Region People’s Republic of China; 2 Department of Pathology, People’s Hospital of Xinjiang Uygur Autonomous Region, Urumqi, 830002 Xinjiang Uygur Autonomous Region People’s Republic of China; 3 College of Pharmacy, Xinjiang Medical University, Urumqi, 830054 Xinjiang Uygur Autonomous Region People’s Republic of China; 4 Graduate School of Xinjiang Medical University, Urumqi, 830011 Xinjiang Uygur Autonomous Region People’s Republic of China; 5 Clinical Laboratory Center, People’s Hospital of Xinjiang Uygur Autonomous Region, No.91 Tianchi Road, Urumqi, 830002 Xinjiang Uygur Autonomous Region People’s Republic of China

**Keywords:** Docetaxel, Tregs, Exosomes, ZNF667-AS1, Prostate cancer

## Abstract

**Background:**

Docetaxel (DTX) resistance attenuates anti-tumor effects of DTX on prostate cancer (mCRPC) and drug resistance was related to Treg expansion in tumors. ZNF667-AS1 played a suppressing role in various tumors and tumor-derived exosomes carry lncRNAs to participate in tumor progression. Here, the effects of ZNF667-AS1 on malignant characteristics and DTX resistance in PC and the effect and its underlying molecular mechanism of tumor-derived exosomes carrying ZNF667-AS1 on Treg expansion were investigated.

**Methods:**

The identification of exosomes were determined using TEM, NTA and western blot. The abundance of genes and proteins were evaluated using IHC, RT-qPCR, western blot and FISH. Malignant phenotypes of PC cells were evaluated by means of Edu, scratch test, transwell, CCK-8 and flow cytometry. The percentage of CD4^+^CD25^+^Foxp^3+^ Tregs was detected using flow cytometry. The location of ZNF667-AS1 was detected using nuclear-cytoplasmic fractionation. The co-location of ZNF667-AS1 and U2AF1 protein was detected using IF-FISH assay. The interactions among ZNF667-AS1, TGFBR1 and U2AF1 were verified using RNA pull-down, RIP and dual luciferase activity.

**Results:**

ZNF667-AS1 expression in PC samples was lowered, which was negatively relative to poor prognosis and DTX resistance. ZNF667-AS1 overexpression inhibited malignant phenotypes of PC cells, tumor growth and DTX resistance. Besides, DTX resistant cell-derived exosomes expressed lower ZNF667-AS1 expression. Exosomes carrying exogenously high ZNF667-AS1 expression derived PC cells or serum of mice suppressed Treg expansion. On the mechanism, ZNF667-AS1 interacted with U2AF1 to destabilize TGFBR1 mRNA and reduce TGFBR1 expression in CD4^+^T cells.

**Conclusion:**

ZNF667-AS1 suppressed cell growth of PC cells, tumor growth of mice and DTX resistance to PC cells and exogenously high ZNF667-AS1 expression in tumor-derived exosomes destabilized TGFBR1 mRNA and reduce TGFBR1 expression through interacting with U2AF1, thus resulting in attenuated Treg expansion, which was related to DTX resistance.

**Supplementary Information:**

The online version contains supplementary material available at 10.1186/s10020-024-00947-z.

## Introduction

Prostate cancer (PC) has a high morbidity and mortality worldwide in men (Sung et al. 2021). Current therapies for PC mainly include chemoradiotherapy, surgery, androgen deprivation, however, the consequences still dissatisfactory (Nuhn et al. 2019). Docetaxel (DTX) had effective effects on metastatic castration-resistant prostate cancer (mCRPC) (Freedland et al. 2021; Ruiz de Porras et al. 2021). However, DTX resistance severely compromises its clinical efficacy in PC (Colloca et al. 2012). Therefore, finding the cause of DTX resistance and restoring sensitivity to DTX are of great clinical importance.

There are diverse immune cells in tumor microenvironment (TME), but not all immune cells are resistant to tumor progression (Hariyanto et al. 2022). Regulator T cells (Tregs), namely, CD4^+^T cells, which express CD25^+^ and FOXP3, exerting an momentous part in maintaining immune homeostasis (Hariyanto et al. 2022). Massive studies have suggested that Tregs promoted tumor progression through immunosuppression (Wang et al. 2021; Zhang et al. 2022a). Furthermore, Treg expansion was closely related to drug resistance in tumors (Streel et al. 2020). However, the relationship between Treg expansion and DTX resistance to PC cells remains unclear.

Exosomes, secreted by various cells including tumor cells, and carrying abundant substances including long non-coding RNAs (lncRNAs), has been flourishing in the field of cancer (Xu et al. 2022). Tumor-derived exosomes mediate tumor malignant behaviors, chemotherapy resistance, and immune microenvironment through lncRNA delivery (He et al. 2021; Wang et al. 2020). For example, Tumor-derived exosomes carrying LINC01091 facilitated gastric cancer (GC) progression through mediating miRNA-128-3p/ELF4/CDX2 axis (Wang et al. 2023). In this study, we focused on ZNF667-AS1 serving as an inhibitor in multi-cancers, such as, cervical cancer, colorectal cancer (Li et al. 2019; Zhuang et al. 2021). However, the effects of ZNF667-AS1 in PC are in the dark. Moreover, does ZNF667-AS1 carried by exosomes secreted by PC cells affect DTX resistance by affecting Treg expansion? More experimental evidence is needed to explain these questions.

Based on these backgrounds, we first investigated the expression of ZNF667-AS in PC cells, DTX-resistant PC cells and exosomes secreted by PC cells or DTX-resistant PC cells. Then, the effects of exogenous overexpression of ZNF667-AS or/and DTX treatment on PC cell proliferation, migration and invasion, apoptosis, DTX sensitivity to PC cells and tumor growth in mice were explored. Furthermore, how does ZNF667-AS1 carried by exosomes derived from PC cells or mouse serum, in which PC cells were subjected to exogenous overexpression of ZNF667-AS and/or DTX treatment, affect Treg expansion and DTX resistance and what are the downstream molecular regulatory mechanisms of ZNF667-AS1? This are further investigated in this study. We believe that our experimental results could provide a feasible way to restore DTX sensitivity to PC cells.

## Methods

### Clinical sample collection

A total of 42 pairs of tumor tissues and adjacent tissues from PC patients and were collected from People’s Hospital of Xinjiang Uygur Autonomous Region. Radical prostatectomy was done without chemotherapy, radiation, or androgen suppression. The characteristics of patients were presented in Table 1. The collected tissues were kept in liquid nitrogen instantly and restored at − 80 °C. In addition, the blood of DTX-resistant PC patients (35 cases) and DTX-sensitive PC patients (25 cases) who underwent at least 4 cycles of DTX-based chemotherapy but not receive any other therapies were collected from People’s Hospital of Xinjiang Uygur Autonomous Region. Blood samples were centrifuged at 1500 × *g* for 10 min at 4 °C, and the serum was collected and stored at − 80 °C. Of note, DTX resistance was diagnosed based on an increasing prostate-specific antigen (PSA) level or radiographic cancer progression during docetaxel administration. All patients enrolled in this study signed the informed consent. Our study was permitted by the ethics committee of People’s Hospital of Xinjiang Uygur Autonomous Region.

**Table 1:** Correlation between lncRNA ZNF667-AS1 expression and clinical pathological characteristic patients with prostate cancer (n = 42)

Parameters	Number	lncRNA ZNF667-AS1		*p* value
		Low (n = 21)	High (n = 21)	
Age (years)				
< 60	20	9	11	0.537
≥ 60	22	12	10	
Tumor size (cm)				
< 2.5	17	7	10	0.346
≥ 2.5	25	14	11	
Gleason score				
< 7	22	8	14	0.064
≥ 7	20	13	7	
Lymph-node metastasis				
Negative	19	6	13	0.042*
Positive	23	15	8	
Clinical stage				
I-II	25	9	16	0.047*
III-IV	18	12	6	

^*^P < 0.05

### Quantitative real-time PCR (RT-qPCR)

To evaluate targeted gene expression in samples of tissues and blood from PC patients, tumor tissues from PC mice, WPMY-1 cells, PC cells, CD4^+^T cells and PC cells-derived exosomes. RT-qPCR was conducted. In this experiment, the primers of ZNF667-AS1, transforming growth factor beta receptor 1 (TGFBR1) and GAPDH were presented in Table 2, which were synthesized by Sangon (China). TRIzol reagent (Beyotime, China) and Script Reverse Transcription Reagent Kit (TaKaRa, China) were respectively used for extraction of RNA from samples and synthesis of cDNA. SYBR Premix Ex Taq II Kit (TaKaRa) was used for qPCR process. 2^−ΔΔCt^ formula was used to compute the relative expression of genes. GAPDH served as a reference gene.

**Table 2:** Primer sequences for RT-qPCR

Gene	Forward (5′-3′)	Reverse (5′-3′)
ZNF667-AS1	AGACTGGACCCTGCTACGAT	CAGGGGCACTACTGCTGAAA
TGFBR1	TGCCATAACCGCACTGTCA	AATGAAAGGGCGATCTAGTGATG
GAPDH	AGCCCAAGATGCCCTTCAGT	CCGTGTTCCTACCCCCAATG

### Fluorescence in situ hybridization (FISH)

FISH assay was conducted to examine ZNF667-AS1 expression in tumor tissues of DTX-resistant and DTX-sensitive PC patients. In short, ZNF667-AS1 probe was synthesized by Servicebio (China), which was used for incubate samples in this study. Afterwards, DAPI was applied to stain cell nuclei in tissues. Finally, using a fluorescence microscope (Olympus, Japan), fluorescence intensity of samples was examined and images were obtained.

### Cell culture and treatment

Human PC cell lines including PC-3, DU145, VCaP, 22RV1 and LNCaP cells and normal prostatic epithelial cell line (WPMY-1), and murine PC cell line (RM-1) were acquired from American Type Culture Collection (ATCC, USA). RPMI 1640 medium (Thermo Fisher Scientific, USA), which were added with 10% FBS (Thermo Fisher Scientific) and 1% antibiotics (Beyotime, China), was used for incubation of above-mentioned cells. Cells were placed in incubator under 5% CO_2_ and at 37 °C. All cells were performed short tandem repeat (STR) authentication, confirming no misidentification or contamination (IGE Biotechnology, Guangdong, China).

According to a previous study (O'Neill et al. 2011), DTX-resistant PC-3/DU145 cells were established through stepwise increased concentrations of DTX (Cayman chemical Company, USA) up to a final concentration of 10 nM. Of note, DTX-resistant cells were regularly cultured in medium containing 10 nM DTX. For experiments involving DTX intervention cells, the selected DTX concentration was 4 nM and the intervention time was 48 h.

### Cell transfection

To overexpress ZNF667-AS1 and silence ZNF667-AS1, lentivirus carrying overexpression vector of ZNF667-AS1 (ov-ZNF667-AS1), short hairpin RNA targeting ZNF667-AS1 (sh-ZNF667-AS1) were applied to infect PC cells and CD4^+^ T cells. In addition, to knock down U2 small nuclear RNA auxiliary factor 1 (U2AF1), small interfering RNA targeting U2AF1 (si-U2AF1) was transfected into CD4^+^ T cells using Lipofectamine^™^ 3000 (Invitrogen, USA) following the instructions. Lentivirus carrying ov-ZNF667-AS1 or sh-ZNF667-AS1 and si-U2AF1 as well as its control were obtained from GenePharma (China).

### 5-Ethynyl-2′-deoxyuridine (Edu) assay

Edu assay was employed to detect cell proliferation of PC cells with indicated treatments. In short, PC cells were seeded in 96-well plates at the density of 1 × 10^3^ cells/well. After overnight growth, according to tutorial manual, cell proliferation was measured using BeyoClick™EDU-488 kit (Beyotime, China). After being treated with a kit, the stained cells were pictured by a microscope and EdU positive cells was calculated using ImageJ.

### Scratch test

Scratch test was applied to evaluate cell migration of PC cells with indicated treatments. In short, PC cells were implanted on 6-well plates (3 × 10^5^ cell/well) with cellular monolayers overnight. Then, cells were scratched with a micropipette tip. After washed using PBS solution, cells were continually cultured for 24 h. The scratched widths were measured under an optical microscope and recorded at 0 and 24 h.

### Transwell

Transwell assay was used for evaluation of cell invasion of PC cells. Shortly, a 24-well transwell insert system (Corning, USA) was used in this study. Top chamber of transwell insert system was covered with Matrigel (Becton Dickinson Biosciences, USA). PC cells with indicated treatments were inoculated onto top chamber (1 × 10^5^ cell/well), which were added with serum-free RPMI 1640 medium. Additionally, RPMI 1640 medium containing 10% FBS was added into lower chamber of transwell insert system. After 24 h, invaded cells, which was located on the below side of chamber, underwent fixation with 95% alcohol and stained with 1% crystal violet (Sigma-Aldrich, USA). An optical microscope was used for observation of invaded cells.

### Cell counting kit-8 (CCK-8)

CCK-8 assay was used to detect cell viability of PC cells with indicated treatments. PC cells were implanted onto 96-well plates (1 × 10^3^ cells/well) and cultured for 24 h. Subsequently, 10 μL CCK8 solution (Beyotime) was supplemented into cells of each well for 2 h incubation. Then, absorbance value of each well was measured by a microplate reader (Thermo Fisher Scientific) when wave length was regulated at 450 nm.

### Flow cytometry

Flow cytometry was employed to examine cell apoptosis of PC cells with indicated treatments. Shortly, collected cells were cultured with 10 μL Annexin V-FITC and 5 μL PI stain in the dark. After incubation for 10 min, cells were immediately analyzed using flow cytometry (BD Biosciences, USA).

In addition, flow cytometry was applied to detect the percentage of CD4^+^CD25^+^Foxp3^+^ T cells. The CD4^+^CD25^+^Foxp3^+^ Tregs staining kits (Invitrogen, USA) were employed to stain the collected CD4^+^ T cell populations following manufacturer’s instructions. After washing, cells were analyzed by a flow cytometer.

### The isolation and identification of exosomes

For exosomes isolation, the supernatants of PC cells and serum of mice were collected after ultracentrifugation. Transmission electron microscopy (TEM) and nanoparticle tracking analysis (NTA) were employed for observing the structure of exosomes and measuring the particle sizes. Western blot was applied to detect the markers CD63(PA5-92370, 1:1000, Thermo Fisher Scientific), TSG101 (ab125011, 1:5000, Abcam, UK) and Alix (ab275377, 1:1000, Abcam) of exosomes, which were used to identify exosomes.

### Immunohistochemical (IHC) experiment

IHC experiment was performed to determine FOXP3 expression in clinical specimens. Shortly, after fixation with 4% PFA, embedding with paraffin, around 5 μm sections were prepared. Then, sections were subjected to repairing the antigen. Then, sections were blocked with 1% BSA. Afterwards, anti-FOXP3 (Abcam) was used to incubate sections overnight at 4 °C. Next, HRP-labelled antibody (Abcam) and diaminobenzidine (DAB) were used to incubate sections in turn. Finally, using an optical microscope (Olympus, Japan), the images were observed and taken.

### The isolation of CD4^+^ T cells

Fresh spleens and peripheral blood of mice with indicated treatments were immediately homogenized. A CD4^+^ T cell isolation kit (Miltenyi Biotec, Germany) was applied to purify lymphocyte subsets following manufacturer’s instructions. CD4^+^ T cells were cultured in RPMI 1640 medium supplemented with 1 mM HEPES, 1 mg/mL anti-mouse CD3e (BD Biosciences), 1 mg/mL anti-mouse CD28 (BD Biosciences), and 12% FBS in a 5% CO_2_ atmosphere.

### Exosomes uptake with PKH26 labeling

To investigate whether CD4^+^ T cells could take up exosomes, a PKH26 red fluorescent cell linker kit (Sigma-Aldrich) was used to label exosomes. Then, the labeled exosomes were added to CD4^+^ T cells and incubated for 24 h. Besides, the nuclei of CD4^+^ T cells were stained with DAPI. A fluorescence microscope (Leica, Germany) was employed to observe the uptake of labeled exosomes by CD4^+^ T cells.

### Animal experiments

Animal experiments were approved by the Ethics Committee of People’s Hospital of Xinjiang Uygur Autonomous Region. A total of 60 C57BL/6 J mice (Beijing Vital River Laboratory Animal Technology Co. Ltd) were divided into six groups (10 mice/group) randomly. The detailed groups were as follows: Control, DTX, sh-ZNF667-AS1, DTX+sh-ZNF667-AS1, ov-ZNF667-AS1 and DTX+ov-ZNF667-AS1. RM-1 cells (about 2 × 10^6^ cells) infected with lentivirus carrying ov-ZNF667-AS1, sh-ZNF667-AS1, were inoculated into the subcutaneous axilla of the left forelimb of mice to establish a model of tumor formation. On day four post inoculation, DTX solution (10 mg/kg) was intravenously injected into partial mice every 3 d for 4 consecutive injections. Tumor volume was then measured every 7 days until 35 days. Then, mice were euthanized and tumor tissues, peripheral blood and spleen were collected for subsequent experiments.

### Nuclear-cytoplasmic fractionation

Nuclear-cytoplasmic fractionation was employed to assess the distribution of ZNF667-AS1 in CD4^+^ T cells. Following, Nuclear and cytoplasmic RNAs of CD4^+^ T cells were separated using PARIS™ Kit (Invitrogen, USA). The expression of ZNF667-AS1 in cytoplasm and nucleus of CD4^+^ T cells was detected by RT-qPCR.

### RNA immunoprecipitation (RIP) assay

RIP assay was conducted to verify the interaction between ZNF667-AS1 and U2AF1 protein in CD4^+^ T cells. In short, CD4^+^ T cells (around 1 × 10^7^ cells) were harvested and lysed using RIP lysis buffer for 5 min. were lysed with RIP lysis buffer. Supernatants were collected after centrifugation. Magnetic beads conjugated with anti-U2AF1 (ab172614, Abcam) or Ig G (ab172730, Abcam) were added to the supernatants. IgG acted as control. The RNA complexes bound to the beads were eluted with elution buffer. The immunoprecipitated RNAs were determined using RT-qPCR.

### RNA pull-down

RNA pull-down was used to validate the interaction between ZNF667-AS1and U2AF1 and the interaction between ZNF667-AS1 and TGFBR1 mRNA in CD4^+^ T cells. In brief, CD4^+^ T cells were transfected with a biotin-labelled probe against ZNF667-AS1. 48 h later, the cells (around 1 × 10^7^ cells) were washed and incubated with lysis buffer after washing. The lysate was incubated with streptavidin-coated magnetic beads at overnight 4 °C. The enrichment of U2AF1 protein and TGFBR1 mRNA was measured by western blot and RT-qPCR.

### IF-FISH assay

According to a previous study (Zeng et al. 2018), we conducted an IF-FISH assay to detect co-location of ZNF667-AS1 and U2AF1 protein. Briefly, CD4^+^ T cells were fixed with 4% paraformaldehyde and permeabilized with 0.5% Triton X-100. Then, the cells were then incubated with anti-U2AF1 (ab172614, Abcam) and fluorescence-labelled secondary antibody. After the antibody incubation, CD4^+^ T cells were fixed with 4% paraformaldehyde and dehydrated in 70%, 95%, and 100% ethanol. The dehydrated cells were then hybridized with ZNF667-AS1 probe. Confocal images were captured using a fluorescence microscope (Carl Zeiss, USA).

### Dual luciferase activity experiment

Dual luciferase activity experiment was employed to validate the interaction between ZNF667-AS1 and TGFBR1. Briefly, TGFBR1 3’-UTR wild type (TGFBR1-WT) and mutant (TGFBR1-MUT) were obtained from GenePharma. Ov-ZNF667-AS1/ov-NC in combination with TGFBR1-WT/TGFBR1-MUT were transfected into CD4^+^ T cells using Lipofectamine 3000 (Invitrogen, USA). A dual luciferase reporter system kit (Promega, USA) was employed to assess luciferase activity.

### The detection of RNA stability

To determine how ZNF667-AS1 and U2AF1 affects the RNA stability of TGFBR1 mRNA in CD4^+^ T cells, CD4^+^ T cells were transfected with ov-ZNF667-AS1 or together with si-U2AF1 and then 2 μg/mL actinomycin D was used to block gene transcription process in CD4^+^ T cells. For TGFBR1 mRNA stability, RT-qPCR was used to detect the level of TGFBR1 mRNA in CD4^+^ T cells after actinomycin D for 0, 4, 8, 12, 24 h.

### Western blot

Total proteins were obtained from exosomes derived from PC-3 and DU145 cells, PC-3/DTX and DU145/DTX cells and CD4^+^ T cells using RIPA lysis buffer (Beyotime). Then, the concentration of proteins was determined using the BCA protein concentration determination kit (Beyotime). Proteins were isolated using electrophoresis. Afterwards, isolated proteins were transferred onto PVDF membranes. After blocking by skimmed milk (5%), membranes were incubated overnight at 4 °C with primary antibodies including TGFBR1 (ab235578, 1:1000), U2AF1 (ab172614, 1:3000) and β-actin (ab8226, 1:1000) purchased from Abcam. Then, after washing the membranes with Tris–Borate-Sodium Tween-20 (TBST) solution, membranes were immersed in HRP-conjugated secondary antibody (Beyotime) for 1 h. Finally, protein bands were visualised using an ECL kit (Beyotime) and the grey values of protein bands were evaluated by ImageJ.

### Statistical analysis

Statistical analysis of all data and pictures were conducted using SPSS21.0 (SPSS Inc., USA) and GraphPad Prism 9.0 (GraphPad Software Inc., USA). Shapiro–Wilk test was used to test the normal distribution and the measurement data of the normal distribution were in the form of mean ± standard deviation (SD). If comparing between two groups, unpaired Student’s t-test was adopted to analyze data. If three groups or more were compared, one-way analysis of variance (ANOVA) with Tukey’s post hoc test were chosen. Survival time of patients and mice was analyzed using Kaplan–Meier. The relationship between ZNF667-AS1 and TGFBR1 were analyzed using Pearson correlation analysis. *p* < 0.05 was regarded as statistically significant difference.

## Results

### Down-regulated expression of ZNF667-AS1 in PC tissues was positively correlated with poor prognosis and DTX resistance

**Fig. 1:**
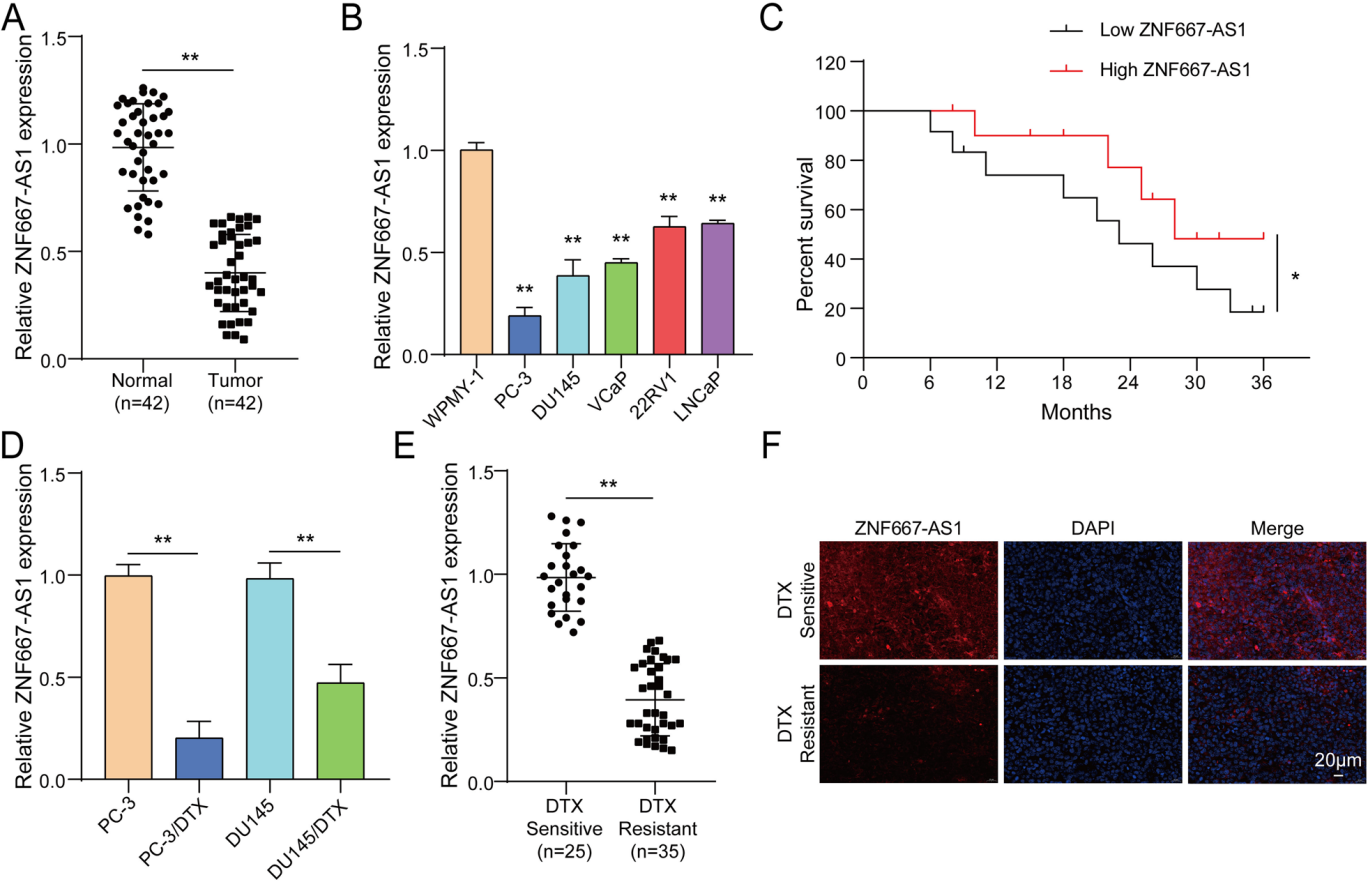
Down-regulated expression of ZNF667-AS1 in PC tissues was positively correlated with poor prognosis and DTX resistance. **A** ZNF667-AS1 expression was detected in tumor tissues and para-carcinoma tissues from 42 PC patients using RT-qPCR. **B** ZNF667-AS1 expression was examined in WPMY-1 cells and several PC cell lines (PC-3, DU145, VCaP, 22RV1 and LNCaP) using RT-qPCR. **C** The relationship between ZNF667-AS1 expression and survival time in PC patients was analyzed using Kaplan–Meier. **D** ZNF667-AS1 expression was determined in PC cells (PC3, DU145) and DTX-resistant PC cells (PC3/DTX, DU145/DTX) using RT-qPCR. **E** ZNF667-AS1 expression was analyzed in blood samples of DTX-resistant PC patients and DTX-sensitive PC patients using RT-qPCR. **F** ZNF667-AS1 expression was evaluated in tumor tissues from DTX-resistant PC patients and DTX-sensitive PC patients using FISH assay. *P < 0.05, **P < 0.01

Tumor tissues and para-carcinoma tissues from 42 PC patients were collected. As presented in Fig. 1A, ZNF667-AS1 expression was lower in tumor tissues. Similarly, ZNF667-AS1 expression was largely decreased in 5 PC cell lines including PC-3, DU145, VCaP, 22RV1 and LNCaP compared to WPMY-1 cells **(**Fig. 1B**)**. From Table 1, we learned that downregulated ZNF667-AS1 expression in PC patients had a positive related to lymph node metastasis and clinical stage but had no influences on age and tumor size. As expected, lower ZNF667-AS1 expression had an unfavorable overall survival of PC patients **(**Fig. 1C**)**. Furthermore, ZNF667-AS1 expression was lowered in DTX-resistant PC cells (PC3/DTX, DU145/DTX) compared to that in PC cells (PC3, DU145) **(**Fig. 1D**)**. Likewise, ZNF667-AS1 expression was evidently reduced in blood samples from DTX-resistant patients **(**Fig. 1E**)**. Furthermore, FISH assay revealed ZNF667-AS1 expression was evidently declined in DTX-resistant patients **(**Fig. 1F**)**. Collectively, ZNF667-AS1 was lower expression in PC samples, especially in DTX-resistant samples.

### ZNF667-AS1 overexpression suppressed malignant behaviors of PC cells and enhanced DTX sensitivity

**Fig. 2:**
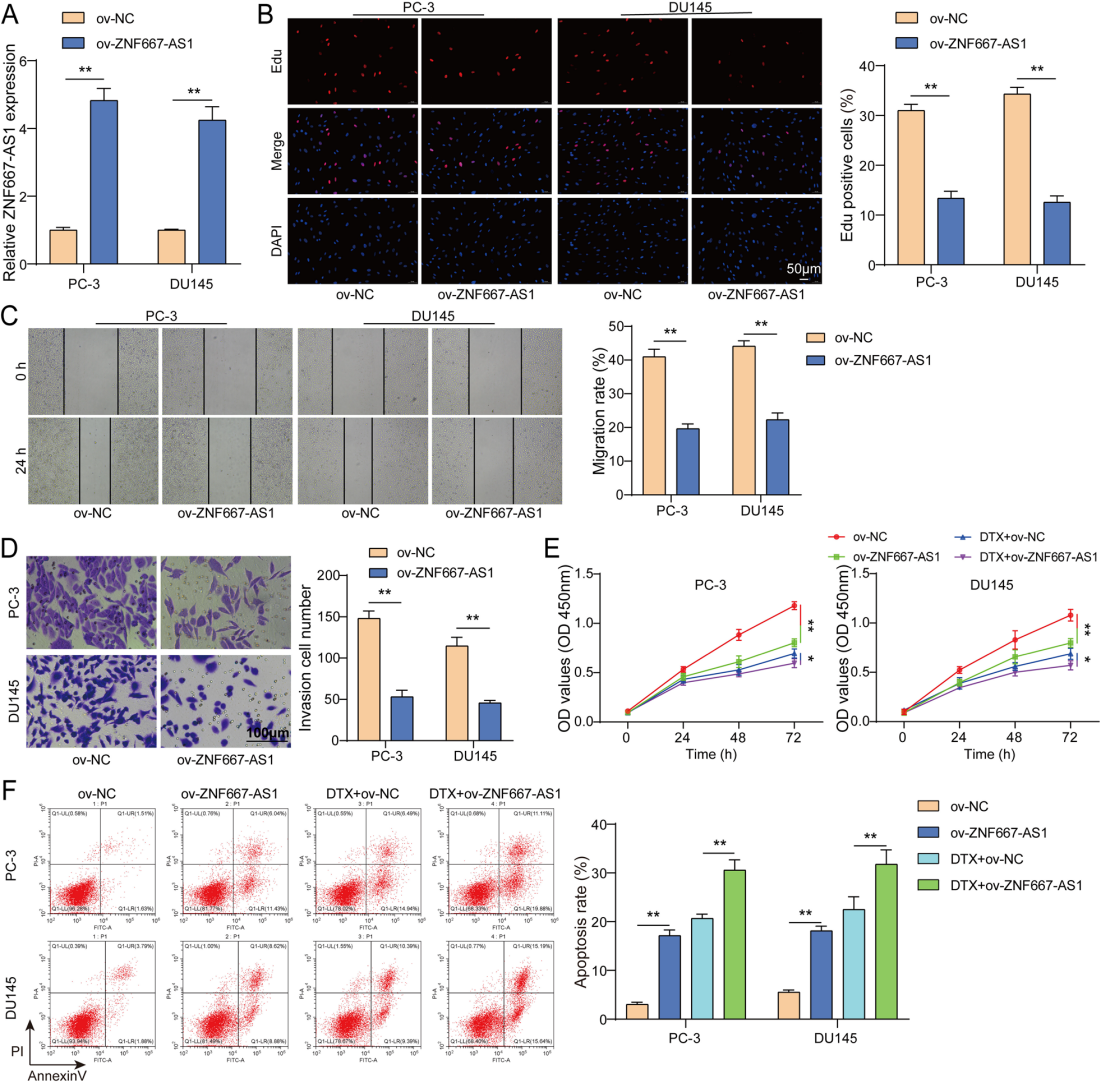
ZNF667-AS1 overexpression suppressed malignant behaviors of PC cells and enhanced DTX sensitivity. PC cells were subjected to ov-NC or ov-ZNF667-AS1 transfection. **A** Transfection efficiency of ov-NC or ov-ZNF667-AS1 transfection into PC cells was evaluated using RT-qPCR. **B** Cell proliferation of PC cells was examined using Edu assay. **C**-**D** Cell migration and invasion of PC cells were measured using scratch test and transwell. PC cells with ov-NC or ov-ZNF667-AS1 transfection were subjected to DTX treatment. **E** Cell viability of PC cells was detected using CCK-8. **F** Cell apoptosis of PC cells was detect using flow cytometry. *P < 0.05, **P < 0.01

According to the results of Fig. 1B, PC-3 and DU145 cells were selected for follow-up experiments due to the lowest expression of ZNF667-AS1. To investigate effects of ZNF667-AS1 on malignant behaviors of PC cells, PC cells were transfected with ov-NC or ov-ZNF667-AS1. Figure 2A exhibited upregulation of ZNF667-AS1 expression in PC cells transfected with ov-ZNF667-AS1 **(**Fig. 2A**)**. Besides, ZNF667-AS1 overexpression suppressed PC cell proliferation, migration and invasion **(**Fig. 2B–D**)**. Additionally, PC cells with ov-NC or ov-ZNF667-AS1 transfection were subjected to DTX treatment. ZNF667-AS1 upregulation enhanced DTX-mediated impairment of PC cell viability and promotion of PC cell apoptosis **(**Fig. 2E, F**)**. Taken together, ZNF667-AS1 upregulation suppressed malignant behaviors of PC cells and elevated DTX sensitivity to PC cells.

### ZNF667-AS1 expression was down-regulated in DTX resistant cells-derived exosomes

**Fig. 3:**
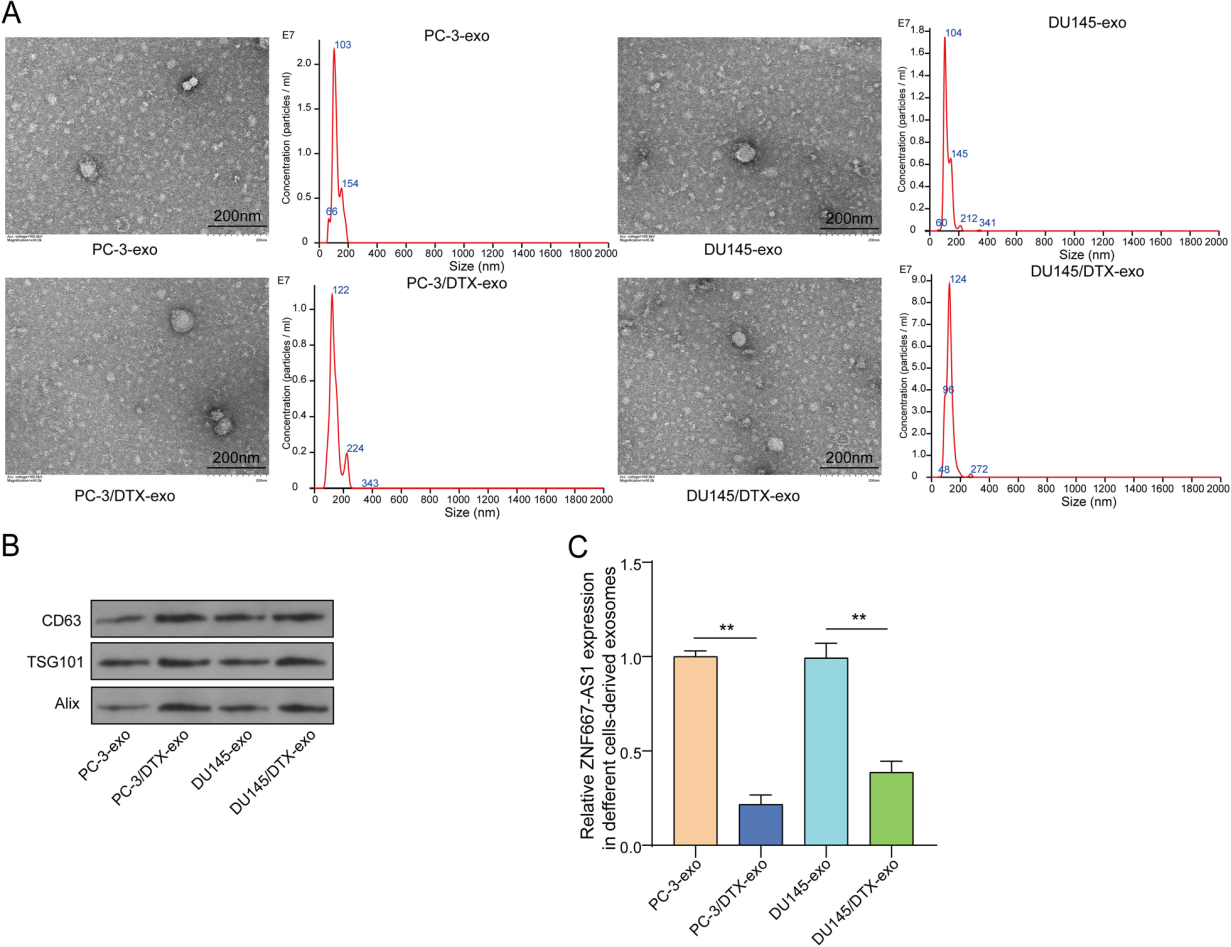
ZNF667-AS1 expression was down-regulated in DTX resistant cells-derived exosomes. Exosomes were isolated from PC-3, DU145, PC-3/DTX and DU145/DTX cells. **A** Morphology of exosomes was observed using a TEM (left). The particle size of exosomes was detected using NTA (right). **B** Exosomes-specific protein markers including CD63, TSG101 and Alix in exosomes was examined using western blot. **C** ZNF667-AS1 expression was evaluated in exosomes using RT-qPCR. **P < 0.01

Tumor cells could secrete exosomes, which participated in regulating diseases (Isaac et al. 2021). Exosomes, secreted by PC-3 cells, DU145 cells, PC-3/DTX cells and DU145/DTX cells, were successfully isolated. TEM and NTA discovered that exosomes were presented the typical rounded, lipid-bilayered particles, with 30–150 nm in diameter **(**Fig. 3A**)**. Exosomes-specific protein markers including CD63, TSG101 and Alix were expressed evidently in the above-mentioned cells-derived exosomes **(**Fig. 3B**)**. Besides, we observed that ZNF667-AS1 expression was largely reduced in PC-3/DTX and DU145/DTX cells-derived exosomes compared to PC cells **(**Fig. 3C**)**. In total, DTX-resistant PC cells-derived exosomes carried lower ZNF667-AS1 expression.

### Exosomes carrying high ZNF667-AS1 expression inhibited Treg expansion

**Fig. 4:**
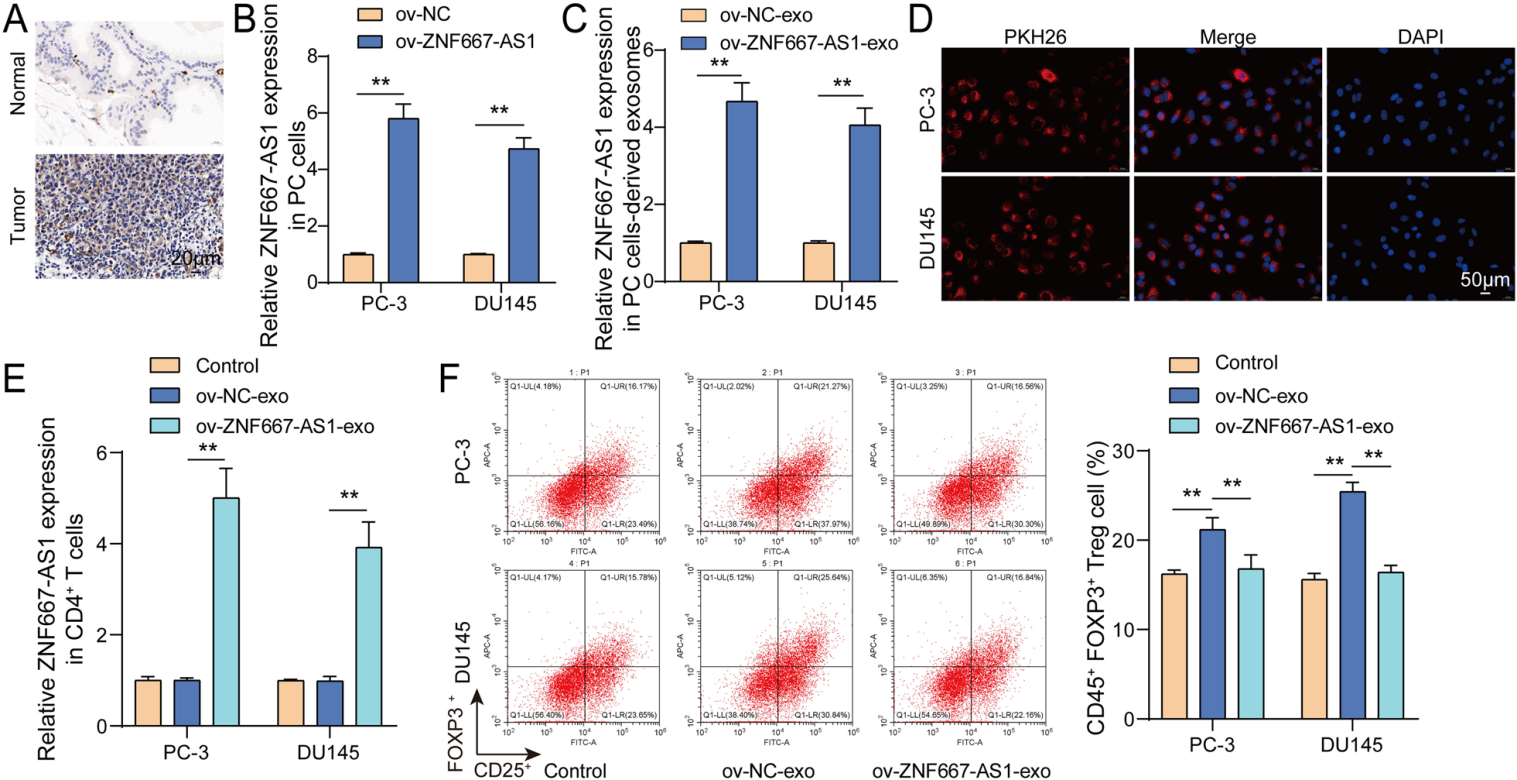
Exosomes carrying high ZNF667-AS1 expression inhibited Treg expansion. **A** FOXP3 expression was measured in tumor tissues and para-carcinoma tissues from PC patients using IHC. **B** Transfection efficiency of ov-NC or ov-ZNF667-AS1 transfection into PC-3 and DU145 cells was evaluated using RT-qPCR. **C** ZNF667-AS1 expression was detected in exosomes derived from PC cells with ov-NC or ov-ZNF667-AS1 transfection using RT-qPCR. **D** The uptake of PKH26-labeled exosomes by CD4^+^ T cells was observed by confocal microscopy. CD4^+^ T cells were treated with exosomes derived from PC cells with ov-NC or ov-ZNF667-AS1 transfection. **E** ZNF667-AS1 expression was examined using RT-qPCR. **F** The percentage of CD4^+^CD25^+^Foxp^3+^ Tregs was detected using flow cytometry. **P < 0.01

Tregs promoted chemotherapy resistance and tumor progression through suppressing tumor immunity (Saleh and Elkord 2019; Zandberg, et al. 2021). As shown in Fig. 4A, FOXP3, a marker of Tregs, was highly expressed in tumor tissues from PC patients **(**Fig. 4A**)**. Subsequently, exosomes were isolated from PC cells transfected with ov-ZNF667-AS1. As anticipated, ZNF667-AS1 expression was enhanced dramatically in PC cells and its-derived exosomes **(**Fig. 4B, C**)**. CD4^+^ T cells ingested successfully the PKH26-labeled exosomes **(**Fig. 4D**)**. Moreover, exosomes derived PC cells with ov-ZNF667-AS1 transfection were used to incubate CD4^+^ T cells for 24 h. ZNF667-AS1 expression in CD4^+^ T cells was upregulated by ZNF667-AS1-overexpressed PC cells-derived exosomes **(**Fig. 4E**)**. PC cells-derived exosomes enhanced the percentage of CD4^+^CD25^+^Foxp^3+^ Tregs, whereas ZNF667-AS1-overexpressed PC cells-derived exosomes compromised the impact of PC cells-derived exosomes **(**Fig. 4F**)**. Collectively, exosomes carrying ZNF667-AS1 overexpression suppressed expansion of CD4^+^ T cells.

### PC tumor-derived exosomes carrying ZNF667-AS1 affected DTX chemotherapy, tumor growth and Treg infiltration

**Fig. 5:**
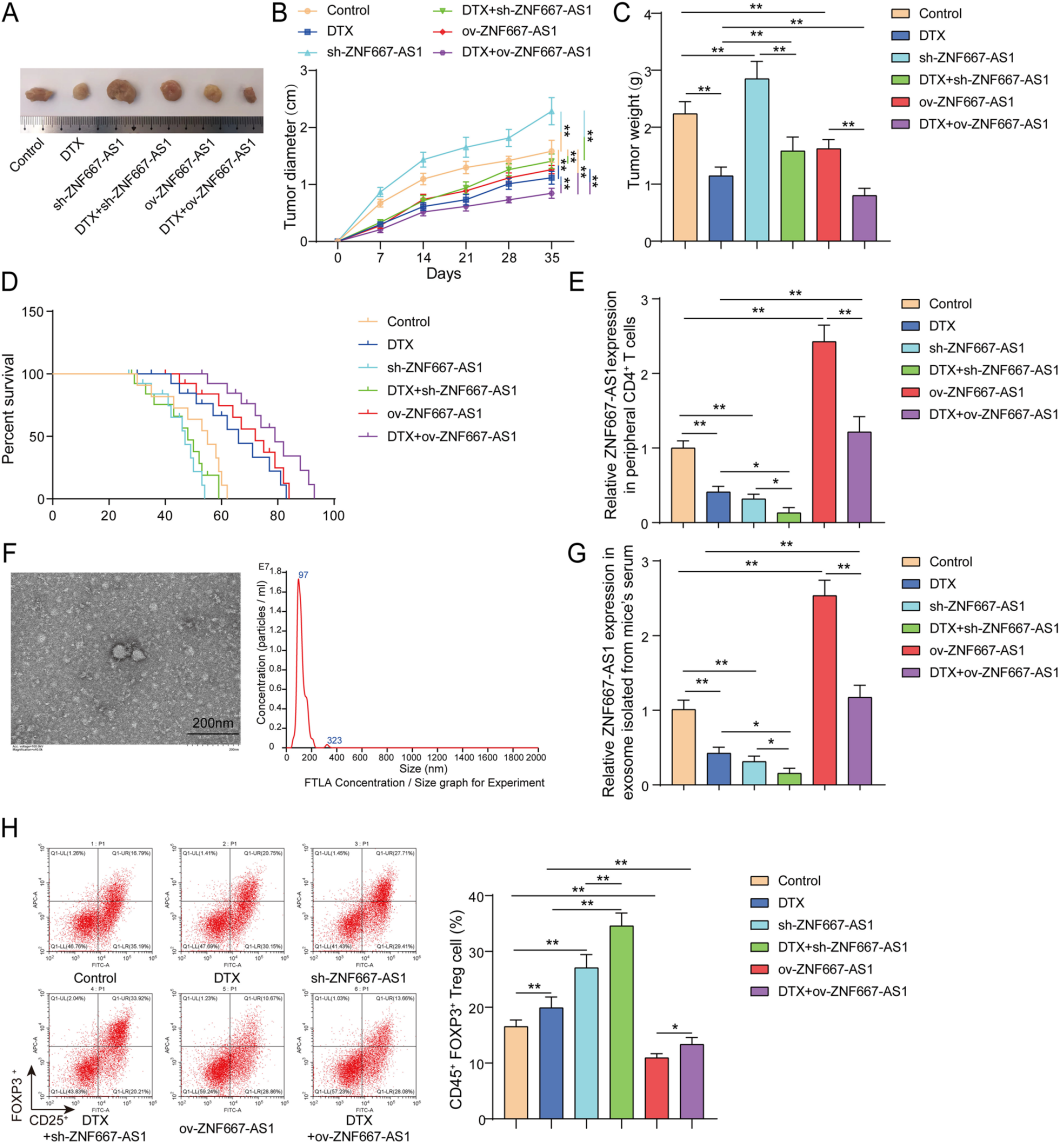
PC tumor-derived exosomes carrying ZNF667-AS1 affected DTX chemotherapy, tumor growth and Treg infiltration. RM-1 cells with sh-ZNF667-AS1 or ov-ZNF667-AS1 transfection were injected subcutaneously into C57BL/6 J mice and some of which were treated with DTX. **A** The pictures of tumors. **B** Tumor weight. **C** Tumor growth curve. **D** Survival time of mice in different groups was analyzed using Kaplan–Meier. **E** ZNF667-AS1 expression was examined in CD4^+^ T cells isolated from peripheral blood of mice using RT-qPCR. **F** The morphology and particle size of exosomes isolated from serum of mice were determined using a TEM and NTA. **G** ZNF667-AS1 expression was detected in exosomes isolated from serum of mice using RT-qPCR. **H** The percentage of CD4^+^CD25^+^Foxp3^+^ Tregs in the total CD4^+^ T cells isolated from peripheral blood and spleen of mice was determined using flow cytometry. *P < 0.05, **P < 0.01

Here, how tumor-secreted exosomes carrying ZNF667-AS1 affect tumor growth and Tregs infiltration in mice were investigated. RM-1 cells were transfected with lentiviruses carrying ov-ZNF667-AS1 or sh-ZNF667-AS1 and then were inoculated into C57BL/6 J mice. As shown in Fig S1A, ZNF667-AS1 expression was evidently elevated in RM-1 cells with ov-ZNF667-AS1 transfection but was apparently reduced in RM-1 cells with sh-ZNF667-AS1 transfection. Furthermore, partial C57BL/6 J mice were subjected to DTX treatment. The detailed groups were Control, DTX, sh-ZNF667-AS1, DTX+sh-ZNF667-AS1, ov-ZNF667-AS1 and DTX+ov-ZNF667-AS1. The results showed that DTX or ZNF667-AS1 overexpression decreased tumor weight and volume and the combination of DTX and ZNF667-AS1 overexpression presented a better inhibition **(**Fig. 5A–C**)**. Inversely, ZNF667-AS1 knockdown greatly promoted tumor weight and volume, whereas DTX treatment impaired these influences of ZNF667-AS1 knockdown **(**Fig. 5A–C**)**. As expected, DTX or/and ZNF667-AS1 overexpression prolonged evidently the survival time of mice, especially the combination of DTX and ZNF667-AS1 overexpression **(**Fig. 5D**)**. However, ZNF667-AS1 knockdown shrank survival time of mice and DTX treatment abolished ZNF667-AS1 knockdown-mediated shortened survival time **(**Fig. 5D**)**. Then, CD4^+^ T cells were isolated from peripheral blood of mice. ZNF667-AS1 knockdown or/and DTX treatment decreased ZNF667-AS1 expression in CD4^+^ T cells, especially the combination of ZNF667-AS1 knockdown and DTX treatment. However, ZNF667-AS1 overexpression increased ZNF667-AS1 expression in CD4^+^ T cells and abolished DTX-mediated reduction of ZNF667-AS1 expression **(**Fig. 5E**)**. Subsequently, exosomes were successfully isolated from serum of mice **(**Fig. 5F**)**. The alterations of ZNF667-AS1 expression in exosomes were similar to changes of ZNF667-AS1 expression in CD4^+^ T cells **(**Fig. 5G**)**. Total CD4^+^ T cells were isolated from peripheral blood and spleen of mice. The percentage of CD4^+^CD25^+^Foxp3^+^ Tregs in total CD4^+^ T cells was elevated apparently by DTX treatment or/and ZNF667-AS1 knockdown, especially the combination of DTX treatment or/and ZNF667-AS1 knockdown. On the contrary, ZNF667-AS1 overexpression decreased the percentage of CD4^+^CD25^+^Foxp3^+^ Tregs and impaired DTX-mediated increased percentage of CD4^+^CD25^+^Foxp3^+^ Tregs **(**Fig. 5H**)**. Taken together, PC tumor-secreted exosomes carrying exogenously high ZNF667-AS1 expression suppressed tumor growth, attenuated DTX resistance and reduced the percentage of Tregs in mice.

### ZNF667-AS1 destabilized TGFBR1 mRNA through interacting with U2AF1

**Fig. 6:**
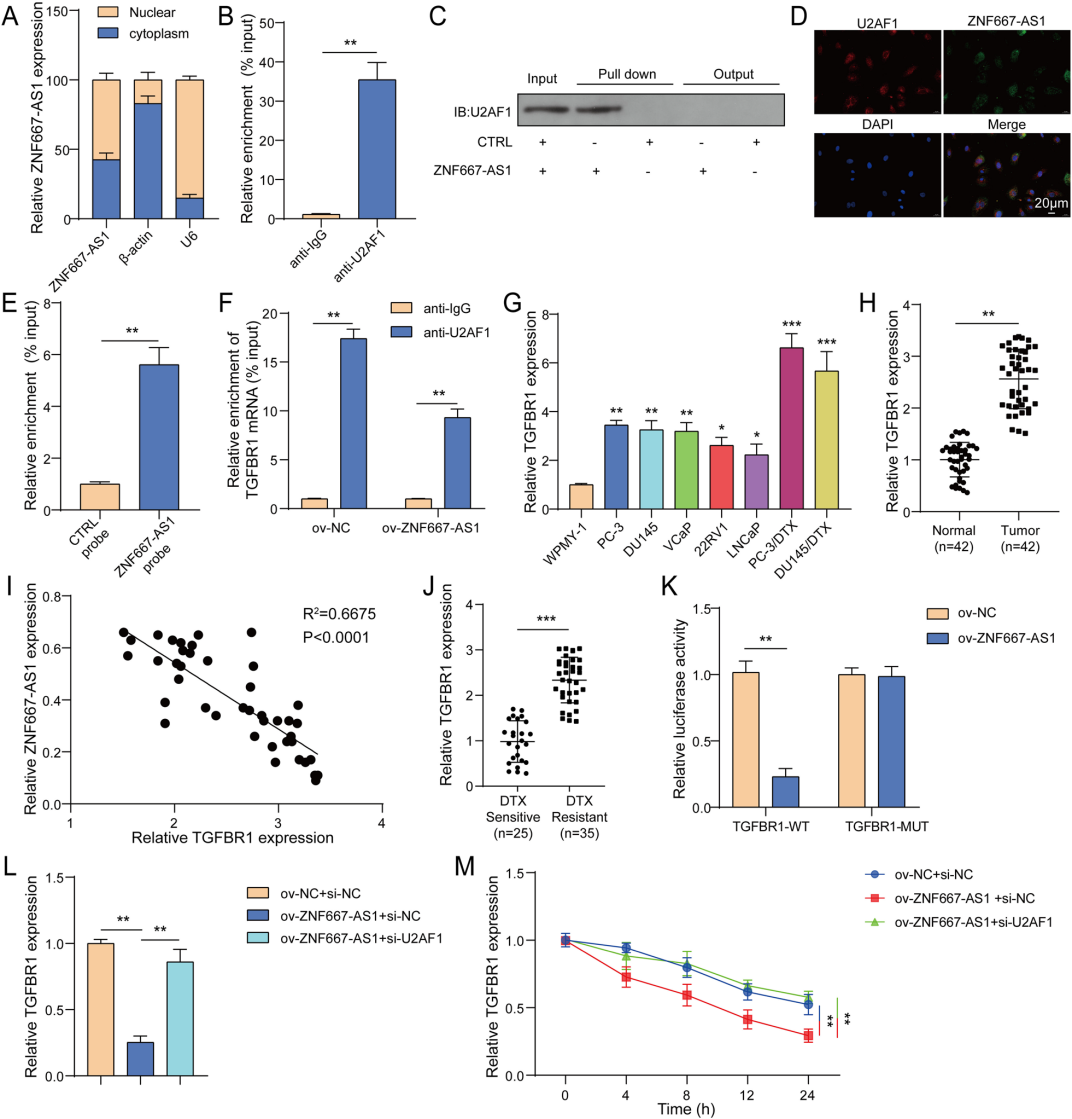
ZNF667-AS1 destabilized TGFBR1 mRNA through interacting with U2AF1. **A** The location of ZNF667-AS1 in CD4^+^T cells was determined using nuclear-cytoplasmic fractionation. **B**-**C** The interaction between ZNF667-AS1 and U2AF1 in CD4^+^T cells was verified using RIP and RNA pull-down. **D** The co-location of ZNF667-AS1 and U2AF1 protein in CD4^+^T cells was detected using IF-FISH assay. **E** The interaction between ZNF667-AS1 and TGFBR1 mRNA in CD4^+^T cells was validated by RNA pull-down. CD4^+^T cells were subjected to ov-NC or ov-ZNF667-AS1 transfection. **F** The interaction between TGFBR1 and U2F1 in CD4^+^T cells was validated using RIP. **G** TGFBR1 expression was measured in WPMY-1 cells, PC cells (PC-3, DU145, VCaP, 22RV1 and LNCaP) and DTX-resistant PC cells (PC3/DTX, DU145/DTX) using RT-qPCR. **H** TGFBR1 expression was measured in tumor tissues and para-carcinoma tissues from 42 PC patients using RT-qPCR. **I** The relationship between ZNF667-AS1 and TGFBR1 expression in PC patients was analyzed using Pearson correlation analysis. **J** TGFBR1 expression in blood of DTX-sensitive patients and DTX-resistant patients was examined by RT-qPCR. **K** The interaction between ZNF667-AS1 and TGFBR1 in CD4^+^T cells was validated by luciferase activity experiment. CD4^+^T cells were transfected with ov-ZNF667-AS1 or together with si-U2AF1. **L** TGFBR1 expression was detected using RT-qPCR. CD4^+^T cells were transfected with ov-ZNF667-AS1 or together with si-U2AF1 and then transfected CD4^+^T cells were treated with 2 μg/mL actinomycin D for 0, 4, 8, 12, 24 h. **M** TGFBR1 mRNA stability was detected using RT-qPCR. *P < 0.05, **P < 0.01 and ***P < 0. 001

The downstream regulatory molecules of exosomes carrying ZNF667-AS1 were investigated. Firstly, nuclear-cytoplasmic fractionation revealed that ZNF667-AS1 were mainly distributed in cytoplasm of CD4^+^T cells **(**Fig. 6A**)**. It was reported that U2AF1 served as an RNA binding protein (RBP) to interact with lncRNA (Du et al. 2023). Here, RIP and RNA pull-down validated the interaction between ZNF667-AS1 and U2AF1 **(**Fig. 6B, C**)**. In addition, IF-FISH assay found that ZNF667-AS1 and U2AF1 overlapped in the cytoplasm of CD4^+^T cells **(**Fig. 6D). Encouragingly, ZNF667-AS1 successfully pulled down TGFBR1 mRNA **(**Fig. 6E). U2AF1 antibody enriched TGFBR1 mRNA and the level of TGFBR1 mRNA enriched by U2AF1 antibody was declined by ZNF667-AS1 overexpression **(**Fig. 6F). TGFBR1 expression was greatly elevated in PC cells, PC3/DTX and DU145/DTX cells, especially in PC3/DTX and DU145/DTX cells (Fig. 6G). Similarly, TGFBR1 expression was enhanced in tumor tissues from PC patients (Fig. 6H). Figure 6I indicated the negative relationship between ZNF667-AS1 and TGFBR1 expression in PC patients. TGFBR1 expression was observably elevated in blood from DTX-resistant patients (Fig. 6J). Luciferase activity experiment validated the interaction between ZNF667-AS1 and TGFBR1 in CD4^+^T cells (Fig. 6K). ZNF667-AS1 overexpression decreased TGFBR1 expression in CD4^+^T cells, while U2AF1 knockdown reversed ZNF667-AS1 overexpression-generated reduction of TGFBR1 expression (Fig. 6L). Additionally, ZNF667-AS1 overexpression reduced TGFBR1 mRNA stability in CD4^+^T cells, whereas U2AF1 knockdown abolished ZNF667-AS1 overexpression-mediated this impact (Fig. 6M). Collectively, ZNF667-AS1 attenuated TGFBR1 mRNA stability via interacting with U2AF1.

### TGFBR1 knockdown partially impaired ZNF667-AS1 silencing-induced Tregs expansion

**Fig. 7:**
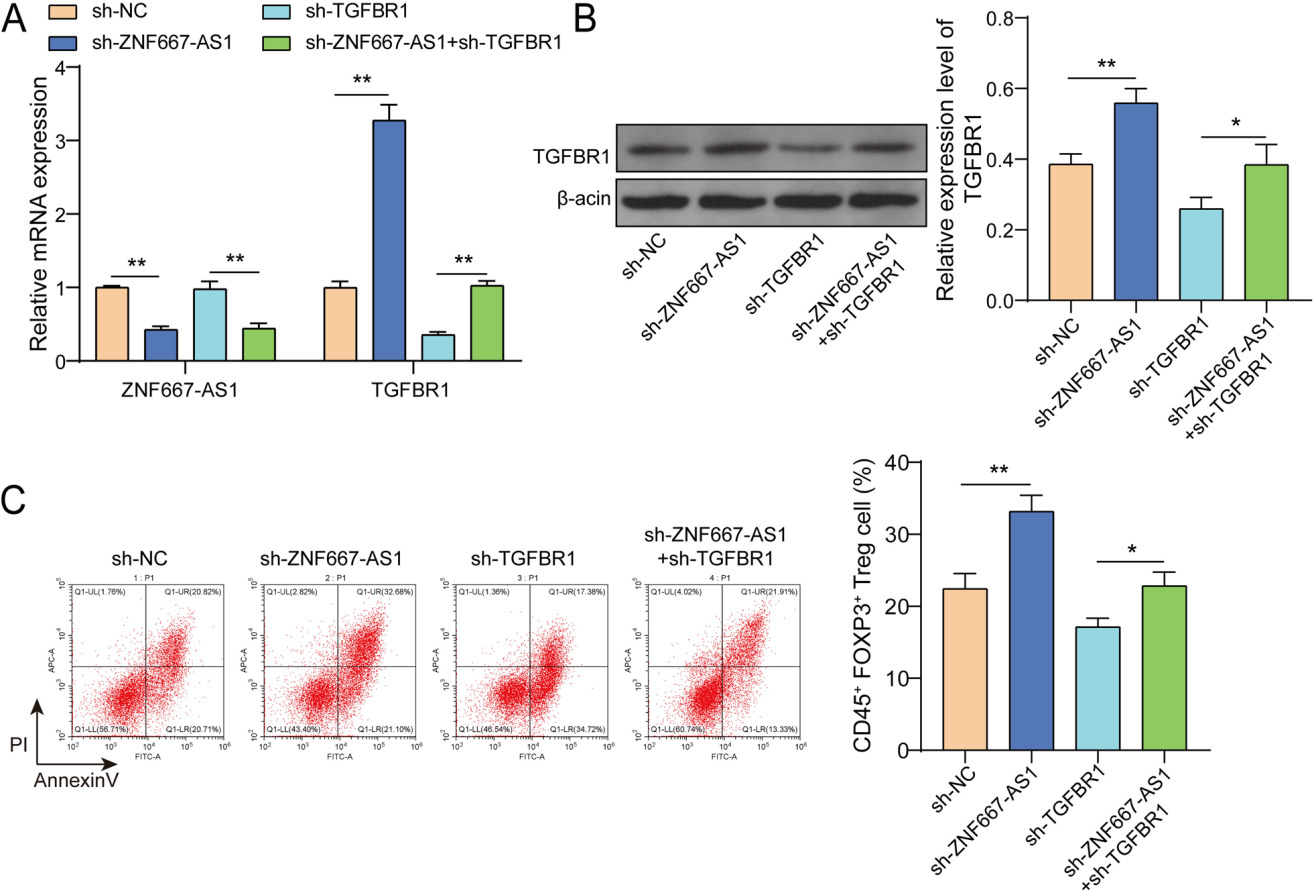
TGFBR1 knockdown partially impaired ZNF667-AS1 silencing-induced Tregs expansion. CD4^+^T cells were transfected with sh-ZNF667-AS1 or/and sh-TGFBR1. **A** ZNF667-AS1 and TGFBR1 expression was detected by RT-qPCR. **B** TGFBR1 expression was measured by western blot. **C** The percentage of CD4^+^CD25^+^Foxp3^+^ Tregs was determined using flow cytometry. *P < 0.05, **P < 0.01

To further clarify whether ZNF667-AS1 affects Tregs expansion via downregulating TGFBR1 expression, CD4^+^T cells were transfected with sh-ZNF667-AS1 or/and sh-TGFBR1. ZNF667-AS1 expression was reduced and TGFBR1 expression was elevated by ZNF667-AS1 knockdown, but TGFBR1 knockdown merely reduced TGFBR1 expression and compromised ZNF667-AS1 expression-mediated elevation of TGFBR1 expression (Fig. 7A, B). Moreover, ZNF667-AS1 knockdown promoted the percentage of CD4^+^CD25^+^Foxp^3+^ Tregs, and TGFBR1 knockdown observably decreased its percentage (Fig. 7C). As expected, TGFBR1 knockdown partially reversed ZNF667-AS1 knockdown-mediated promotion of Treg expansion (Fig. 7C). Collectively, ZNF667-AS1 silencing enhanced Tregs expansion via decreasing TGFBR1 expression.

## Discussion

**Fig. 8:**
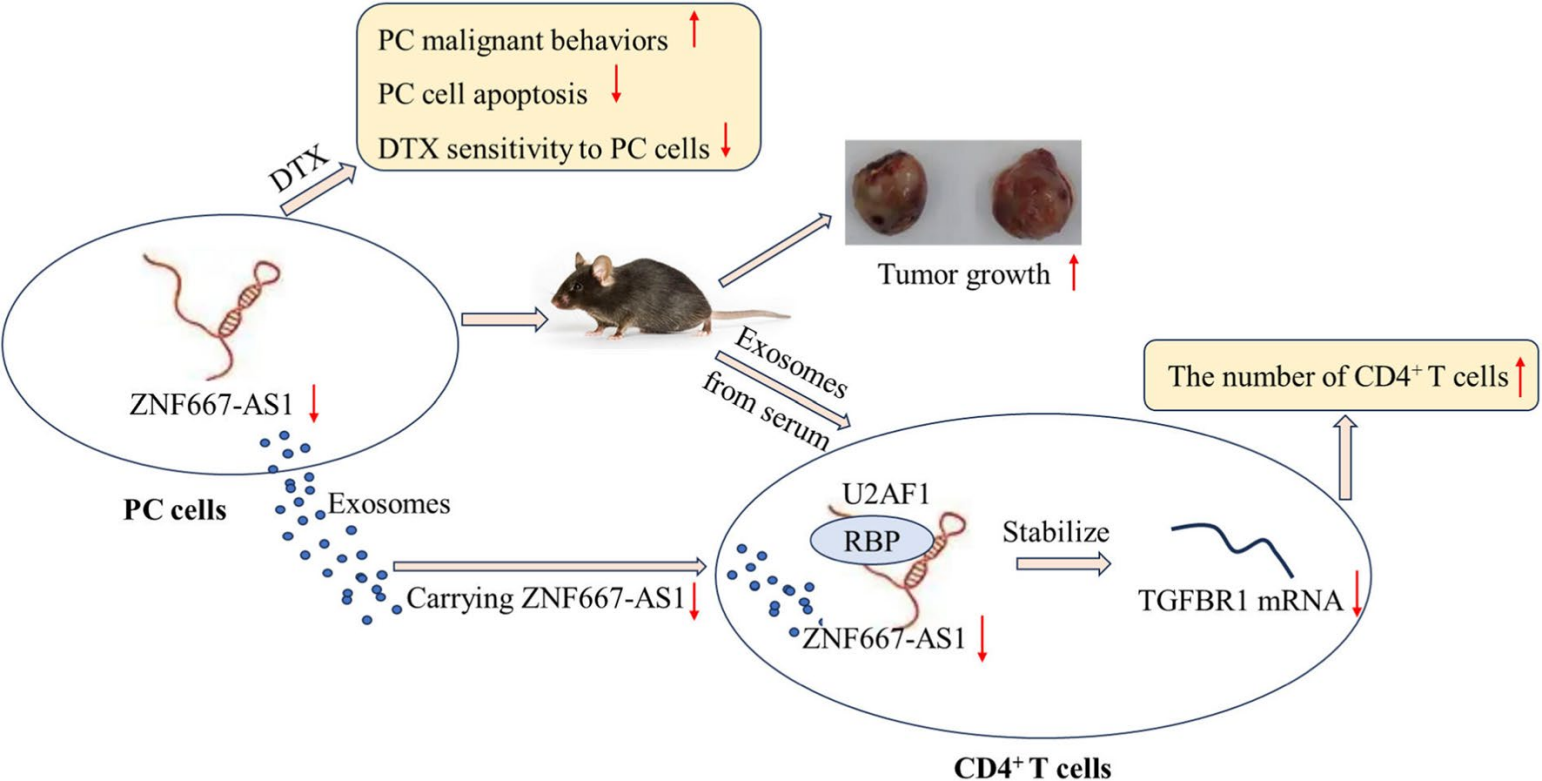
Graphical summary. Illustration of Graphical summary: ZNF667-AS1 expression was lowered in PC cells and PC cell-derived exosomes. Lower ZNF667-AS1 expression in PC cells promoted PC cell proliferation, migration and invasion, reduced PC cell apoptosis, attenuated DTX sensitivity to PC cells and promoted tumor growth in mice. Exosomes carrying lower ZNF667-AS1 expression derived PC cells or serum of mice enhanced TGFBR1 mRNA elevated the number of CD4^+^ T cells through interacting with RBP U2AF1

DTX resistance in PC leads to a significant reduction in the tumor-killing effects, which is one of the important reasons for the further development of PC, especially for mCRPC progression (Babasaki et al. 2021). Therefore, restoring DTX sensitivity to PC cells is an effective approach for PC therapy. Immunosuppression was closely related to chemotherapy resistance in tumor therapy (Okita et al. 2005; Zhu et al. 2022a). For example, Dandan Wu et.al. proposed that the percentage of Tregs was enhanced in the peripheral blood of cisplatin and 5-Fu-resistant patients with GC and evidence showed that Tregs promoted 5-Fu resistance in GC cells (Wu and Wang 2022). At present, growing evidence has demonstrated that ZNF667-AS1 played a suppressing role in various cancers (Zhao, et al. 2021; Zheng et al. 2022). In addition, tumor-derived exosomes carrying lncRNAs were widely implicated in tumor progression (Marima et al. 2024). However, the effects of ZNF667-AS1 in PC or PC/DTX cells as well as exosomes derived from PC cells/PC tumors on biological activities of PC cells, tumor growth in mice, DTX resistance and Treg expansion are still unclear. Here, our findings revealed that ZNF667-AS1 was abnormally lowly expressed in PC cells and PC cell-derived exosomes. ZNF667-AS1 overexpression suppressed cell proliferation, migration and invasion of PC cells, enhanced cell apoptosis of PC cells, promoted DTX sensitivity to PC cells and weakened tumor growth in mice. Exosomes carrying exogenously high ZNF667-AS1 expression derived PC cells or serum of mice reduced the number of CD4^+^ T cells through interacting with RBP U2AF1-mediated promotion of TGFBR1 mRNA, thereby enhancing DTX sensibility to PC cells and suppressing PC progression (Fig. 8).

LncRNAs were reported to play vital roles in various diseases (Bridges et al. 2021). ZNF667-AS1 has been investigated in multi-cancers and was demonstrated playing a suppressing role. For instance, Ying-Juan Zheng et.al. revealed that ZNF667-AS1 was evidently reduced in esophageal squamous cell carcinoma (ESCC) tissues and cell lines and its overexpression impaired malignant features of ESCC cells (Zheng et al. 2022). Similarly, ZNF667-AS1 suppressed the progression of colorectal cancer through affecting ANK2/JAK2 axis (Zhuang et al. 2021). However, the expression and role of ZNF667-AS1 in PC have not been reported in published studies. In current study, we found that ZNF667-AS1 expression was downregulated in tumor tissues of PC patients and PC cells, especially in DTX-resistant tissues and PC/DTX cells. As expected, ZNF667-AS1 overexpression weakened malignant behaviors of PC cells and suppressed tumor growth in mice. Massive evidence has indicated that the close relationship between lncRNAs and drug resistance in cancers (Altschuler et al. 2021). As previously described, lncRNA LOC730101 contributed to darolutamide Resistance in PC (Zhou et al. 2024). Here, we observed that NF667-AS1 overexpression could enhance DTX sensibility to PC cells.

As an intercellular communication tool, exosomes could deliver a lot of information including lncRNAs into the ingested cells to exert biological regulation (Kalluri and LeBleu 2020). For example, tumor-derived exosomes delivering ElNF1-AS1 promoted M2 polarization of macrophages to accelerate GC progression (Ma et al. 2023). Besides, tumor-derived exosomes carrying lincRNA ROR stimulated angiogenesis to facilitate nasopharyngeal carcinoma (Zhang et al. 2022b). Shuyao Zhang et.al. suggested that tumor-derived exosomes carrying lncRNA MIAT strengthened paclitaxel resistance to esophageal cancer cells (Zhang et al. 2023). Marta Szajnik et.al. pointed out that tumor-derived exosomes could mediate Treg expansion and promoted biological activities of Tregs in human head and neck squamous cell carcinoma (Szajnik et al. 2010). Furthermore, tumor-secreted miR-208b enhanced Treg expansion and further influenced oxaliplatin chemosensitivity in colorectal cancer (Ning et al. 2021). Therefore, we speculated that PC cells or tumor-derived exosomes carrying ZNF667-AS1 may influence PC malignant characteristics, Treg expansion and DTX resistance. Herein, we successfully isolated PC cells-derived exosomes. ZNF667-AS1 in exosomes was decreased in DTX-resistant PC cells. Besides, ZNF667-AS1 expression was largely increased in exosomes derived from ZNF667-AS1-overexpressed PC cells and exosomes delivering exogenously high ZNF667-AS1 expression significantly inhibited Treg expansion. Furthermore, ZNF667-AS1 overexpression enhanced ZNF667-AS1 expression in CD4^+^T cells isolated from peripheral blood of mice and exosomes isolated from serum of mice. Furthermore, ZNF667-AS1 overexpression reduced the percentage of CD4^+^CD25^+^Foxp3^+^ Tregs in total CD4^+^ T cells isolated from peripheral blood and spleen of mice and offset DTX-mediated elevation of percentage of CD4^+^CD25^+^Foxp3^+^ Tregs. Taken together, tumor-derived exosomes delivering high ZNF667-AS1 expression suppressed Treg expansion and DTX resistance in mice.

By what molecular regulatory mechanism does ZNF667-AS1 in exosomes inhibit the expansion of Tregs? At present, there are various molecular regulatory mechanisms involving pathological situation of lncRNAs, and the regulatory mechanism mediated by the interaction between lncRNA and RBP is a very important type (Shaath et al. 2022). Massive evidence has demonstrated that lncRNAs could influence mRNA stability of genes to participate in multi-diseases through interacting with RBP (Braga, et al. 2023; Ni et al. 2022). For instance, LINC00641 destabilized GLI1 mRNA through interacting with RPB IGF2BP1, thereby suppressing malignant traits of papillary thyroid carcinoma cells (Meng et al. 2023). LncRNA ZFPM2‐AS1 attenuated the stability of ZFPM2 mRNA to inhibit ZFPM2 expression via interacting with RBP UPF1, thus promoting the progression of lung adenocarcinoma progression (Han et al. 2020). Jiannan Du et.al. suggested that U2AF1 served as an RBP, which could be interacted with lncRNA Pnky (Du et al. 2023). In addition, TGFBR1 was widely investigated and was identified as a promoter in the progression of multi-cancers (Kwon et al. 2021; Zhu et al. 2022b). Notably, Wookbong Kwon et.al. reported that TGFBR1 expression, positively regulated by transcription factor ZNF507, was largely increased in mCRPC, contributing to the progression of mCRPC (Kwon et al. 2021). Similarly, in this study, upregulating expression of TGFBR1 in tissues of PC patients and PC cells, especially in DTX-resistant PC patients and PC/DTX cells. TGFBR1 expression was found to be negatively related to ZNF667-AS1 expression in PC patients. Then, through the mechanism that lncRNAs bound to RBP to affect the stability of target gene mRNA, we validated the interactions among ZNF667-AS1, RBP U2AF1, TGFBR1 with a series of experiments in CD4^+^ T cells and ZNF667-AS1 interacted with U2AF1 to attenuate the stability of TGFBR1 mRNA and reduce TGFBR1 in CD4^+^ T cells. Finally, we found that TGFBR1 knockdown could compromise ZNF667-AS1 silencing-mediated promotion of Treg expansion. Taken together, ZNF667-AS1 overexpression decreased the stability of TGFBR1 mRNA and TGFBR1 expression to reduce the expansion of Tregs through interacting with U2AF1.

## Conclusions

In conclusion, our finding revealed that ZNF667-AS1 overexpression suppressed malignant characteristics of PC cells, DTX resistance and tumor growth in mice. Besides, PC cells-derived exosomes delivering exogenously high ZNF667-AS1 expression suppressed the expansion of Tregs. Furthermore, ZNF667-AS1 interacted with U2AF1 to attenuate TGFBR1 mRNA stability and reduce TGFBR1 expression in CD4^+^ T cells, thereby decreasing Treg expansion, which could be responsible for DTX sensibility to some extent.

## Supplementary Information

Additional file 1: Figure S1 Transfection efficiency of ov-ZNF667-AS1 or sh-ZNF667-AS1 in RM-1 cells. (A) ZNF667-AS1 expression was determined in RM-1 cells with ov-ZNF667-AS1 or sh-ZNF667-AS1 transfection using RT-qPCR. *P<0.05, **P<0.01.

## Data Availability

All data generated or analyzed during this study are included in this published article.

## Abbreviations

DTX: Docetaxel
mCRPC: Metastatic castration-resistant prostate cancer
PC: Prostate cancer
DTX: Docetaxel
TME: Tumor microenvironment
Tregs: Regulator T cells
lncRNAs: Long non-coding RNAs
GC: Gastric cancer
PSA: Prostate-specific antigen
RT-qPCR: Quantitative real-time PCR
U2AF1: U2 small nuclear RNA auxiliary factor 1
TGFBR1: Transforming growth factor beta receptor 1
FISH: Fluorescence in situ hybridization
Edu: 5-Ethynyl-2′-deoxyuridine
CCK-8: Cell Counting Kit-8
TEM: Transmission electron microscopy
NTA: Nanoparticle tracking analysis
IHC: Immunohistochemical
DAB: Diaminobenzidine
RIP: RNA immunoprecipitation
SD: Standard deviation
ANOVA: Analysis of variance
RBP: RNA binding protein
ESCC: Esophageal squamous cell carcinoma
