# Supplementary figures and images for: Prostate cancer cell-derived exosomes ZNF667-AS1 reduces TGFBR1 mRNA stability to inhibit Treg expansion and DTX resistance by binding to U2AF1
Source: Mol Med. 2024 Oct 18;30:179. doi: 10.1186/s10020-024-00947-z (PMC11488200; doi:10.1186/s10020-024-00947-z)

A

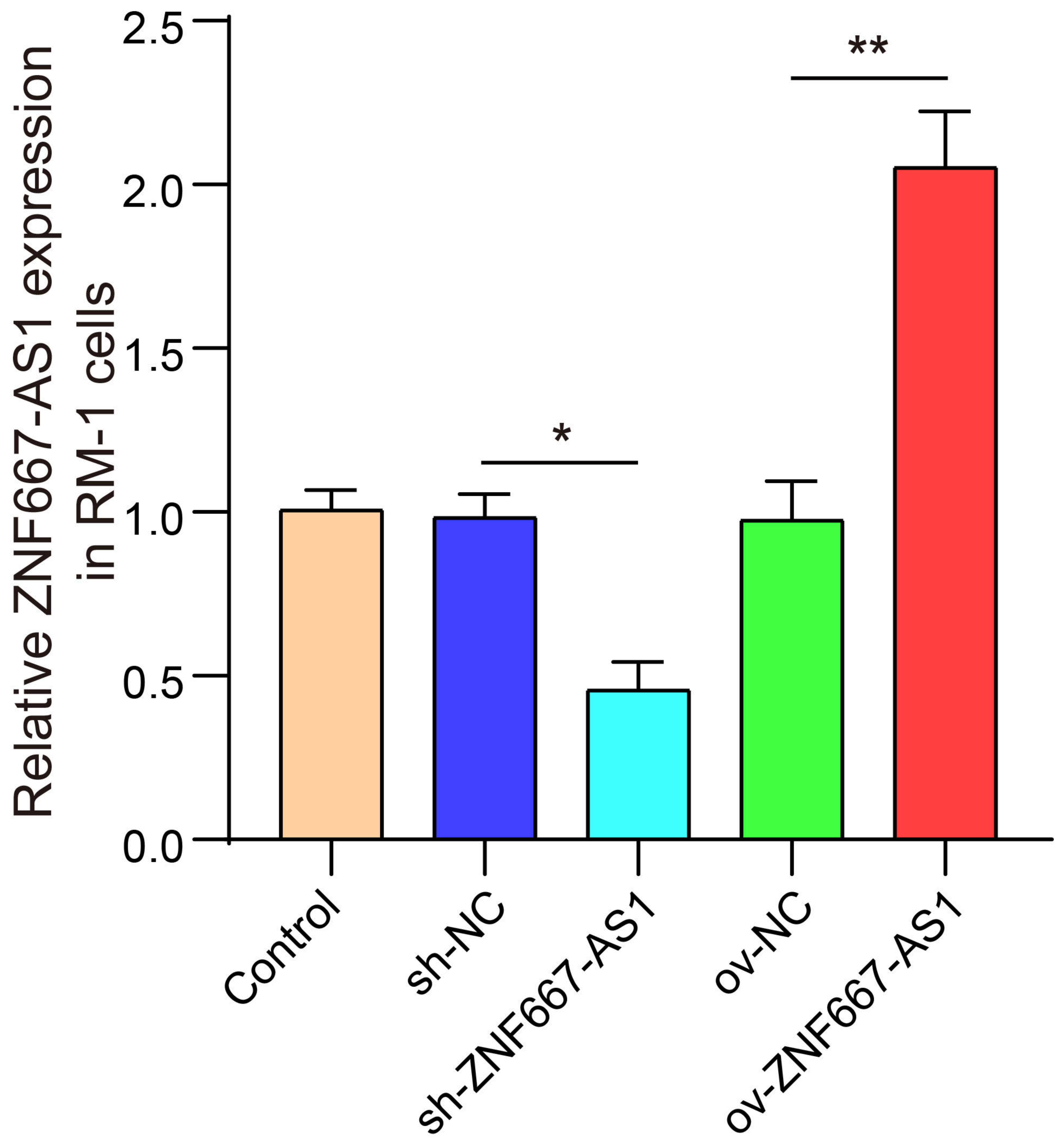

Supplement: Supplementary file 1 — Additional file 1: Figure S1 Transfection efficiency of ov-ZNF667-AS1 or sh-ZNF667-AS1 in RM-1 cells. (A) ZNF667-AS1 expression was determined in RM-1 cells with ov-ZNF667-AS1 or sh-ZNF667-AS1 transfection using RT-qPCR. *P<0.05, **P<0.01. [file 10020_2024_947_MOESM1_ESM.pdf]
